# Crystal structure and DFT study of (*E*)-4-[({4-[(pyri­din-2-yl­methyl­idene)amino]­phen­yl}amino)­meth­yl]phenol

**DOI:** 10.1107/S2056989018003043

**Published:** 2018-02-28

**Authors:** Md. Serajul Haque Faizi, Necmi Dege, Turganbay S. Iskenderov

**Affiliations:** a Department of Chemistry, Langat Singh College, Babasaheb Bhimrao Ambedkar Bihar University, Muzaffarpur, Bihar, India; b Ondokuz Mayıs University, Arts and Sciences Faculty, Department of Physics, 55139 Samsun, Turkey; c Department of Chemistry, Taras Shevchenko National University of Kyiv, 64, Vladimirska Str., Kiev 01601, Ukraine

**Keywords:** crystal structure, Schiff base, pyridine-2-carbaldehyde, amino­phenyl­amino­methyl­phenol, hydrogen bonding, offset π–π inter­actions

## Abstract

In the title Schiff base compound, C_19_H_17_N_3_O, the configuration about the C=N bond is *E*. The mol­ecule is non-planar, with the phenolic and pyridine rings being inclined to the central benzene ring by 56.59 (4) and 15.13 (14)°, respectively. In the crystal, mol­ecules are linked by pairs of O—H⋯N hydrogen bonds, forming inversion dimers. The dimers are connected to neighbouring dimers by N—H⋯O hydrogen bonds and C—H⋯π inter­actions, forming layers parallel to the *bc* plane. The layers are linked by offset π–π inter­actions [inter­centroid distance = 3.779 (2) Å], forming a three-dimensional supra­molecular structure. Quantum chemical calculations of the mol­ecule are in good agreement with the solid-state structure.

## Abstract 2

The title Schiff base compound is considerably non-planar, with the outer phenol and pyridine rings being inclined to each other by 70.21 (3)°.

## Chemical context   

Schiff bases often exhibit various biological activities and, in many cases, have been shown to have anti­bacterial, anti­cancer, anti-inflammatory and anti­toxic properties (Lozier *et al.*, 1975 ▸). Hy­droxy Schiff bases have been studied extensively for their biological, photochromic and thermochromic properties (Garnovskii *et al.*, 1993 ▸; Hadjoudis *et al.*, 2004 ▸). They can be used as potential materials for optical memory and switch devices (Zhao *et al.*, 2007 ▸). Schiff bases derived from pyridine­carbaldehydes have also attracted considerable inter­est in synthetic chemistry. This category covers a diverse range of bidentate or polydentate bridging (Wu & Liang, 2008 ▸; Dong *et al.*, 2000 ▸; Knödler *et al.*, 2000 ▸), which played a significant role in coordination chemistry (Faizi & Hussain, 2014 ▸). Transition metal complexes of pyridyl Schiff bases have found applications in laser dyes (Genady *et al.*, 2008 ▸), catalysis (Wang *et al.*, 2008 ▸) and in crystal engineering, as they form coordination polymers (Huh & Lee, 2007 ▸) or grid-type complexes (Nitschke *et al.*, 2004 ▸). The present work is part of an ongoing structural study of Schiff bases (Faizi *et al.*, 2016 ▸) and their utilization in the synthesis of metal complexes (Faizi & Prisyazhnaya, 2015 ▸). We report herein on the crystal structure and DFT computational calculation of the title Schiff base compound.
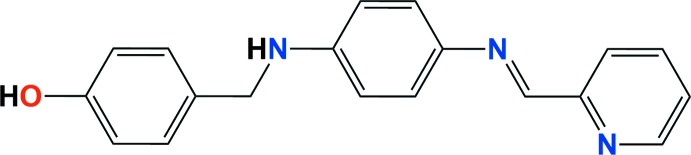


## Structural commentary   

**Figure 1:**
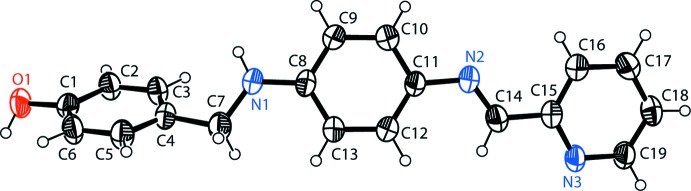
A view of the mol­ecular structure of the title compound, with the atom labelling. Displacement ellipsoids are drawn at the 40% probability level.

The mol­ecular structure of the title compound is illustrated in Fig. 1 ▸. The compound is non-planar; the dihedral angle between the central benzene ring (C8–C13) and the terminal phenolic ring (C1–C6) being 56.60 (13)°. The central benzene ring (C8–C13) is situated in a *trans* position with respect to the terminal pyridine ring (N3/C15–C19); these rings are inclined to each other by 15.13 (14)°. The configuration about the C14=N2 bond is *E*, with a C11—N2—C14—C15 torsion angle of 176.40 (2)°. The C7—N1—C8 angle is 123.43 (1)° and the C7—N1—H1*A*—C8 fragment is approximately planar; the amine N1 atom exhibits a geometry what is typical for an *sp* ^2^ rather than an *sp* ^3^ atom. Bond angles C11—N2—C14 and C15—N3—C19 are also near 120° [121.54 (1) and 117.20 (1)°, respectively], and the imine group has a torsion angle C11—N2—C14—C15 of 176.40 (2)°.

## Supra­molecular features   

**Figure 2:**
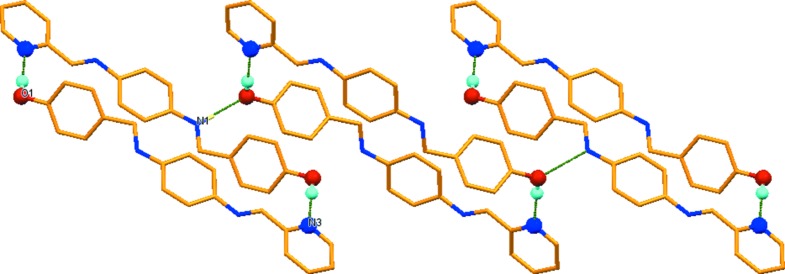
A view along the *b* axis of the inversion dimers, formed *via*. pairs of O—H⋯N hydrogen bonds (thin blue lines), enclosing an 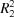
(32) ring motif. The dimers are linked by N—H⋯O hydrogen bonds (see Table 1 ▸ for details).

**Figure 3:**
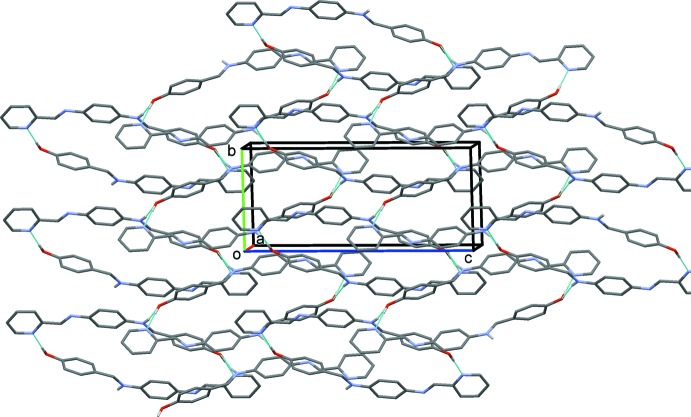
A view along the *a* axis of the layer-like structure in the crystal packing of the title compound. The hydrogen bonds are shown as dashed lines (Table 1 ▸) and only the H atoms involved in hydrogen bonding have been included.

In the crystal, pairs of O—H⋯N hydrogen bonds link the mol­ecules to form inversion dimers, with an 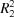
(32) ring motif (Table 1 ▸ and Fig. 2 ▸). The dimers are linked by N—H⋯·O hydrogen bonds (Table 1 ▸ and Fig. 2 ▸) and C—H⋯π inter­actions (Table 1 ▸), forming slabs lying parallel to the *bc* plane (Fig. 3 ▸). The slabs are linked by offset π–π inter­actions involving the pyridine rings, forming a three-dimensional supra­molecular structure [*Cg*⋯.*Cg* ^iii^ = 3.779 (2) Å; *Cg* is the centroid of the N3/C15–C19 ring; inter­planar distance = 3.462 (1) Å and slippage = 1.516 Å; symmetry code (iii) −*x* + 1, −*y* + 2, −*z* + 1].

## Database survey   

A search of the Cambridge Structural Database (Version 5.38, update May 2017; Groom *et al.*, 2016 ▸) for similar structures gave a number of hits for the principal moiety of the title compound, *i.e. N*-(2-pyridyl­methyl­ene)benzene-1,4-di­amine (CSD refcode EXOQAK; Marjani *et al.*, 2011 ▸), and its metal complexes. The pyridine ring in EXOQAK is inclined to the benzene ring by 24.69 (13)° and the adjacent amine and pyridine N atoms are *trans* to each another. In the title compound, the pyridine ring is inclined to the benzene ring by 15.13 (14)° and the N atoms are also *trans* to each another. This is in contrast to the situation in the metal complexes of EXOQAK, *e.g.* di­chloro­{*N*-[(pyridin-2-yl)methyl­ene]benz­ene-1,4-di­amine}­zinc(II) (CSD refcode TUJXIG; Marjani *et al.*, 2009 ▸), where on coordination, the pyridine ring rotates and the adjacent amine and pyridine N atoms are then *cis* to each other.

## DFT study   

The DFT quantum-chemical calculations were performed at the B3LYP/6-311 G(d,p) level (Becke, 1993 ▸) as implemented in *GAUSSIAN09* (Frisch *et al.*, 2009 ▸). DFT structure optimization of (I) was performed starting from the X-ray geometry and the values compared with experimental values (see Table 2 ▸). In general, the calculated values are in good agreement with the experimental data.

**Figure 4:**
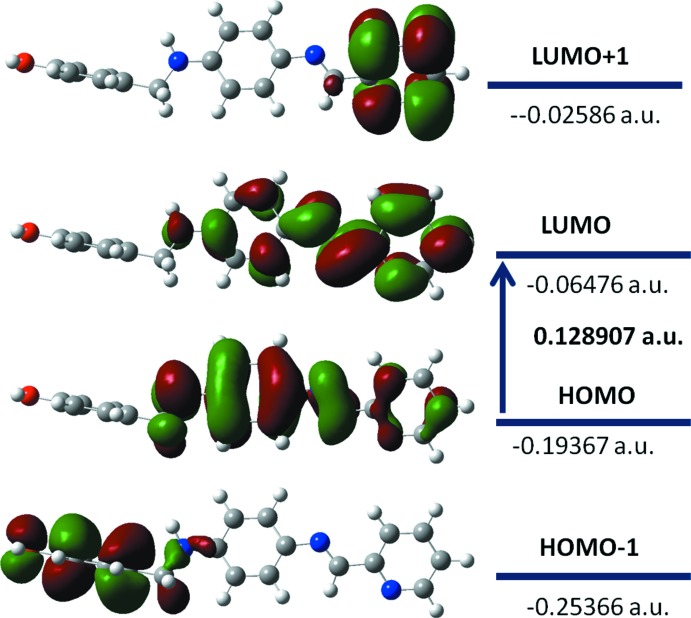
Electron distribution of the HOMO-1, HOMO, LUMO and LUMO+1 energy levels for the title mol­ecule.

The highest occupied mol­ecular orbitals (HOMO) and lowest unoccupied orbitals (LUMO) are named frontier orbitals (FMOs). The LUMO and HOMO orbital energy parameters are considerably answerable for the charge transfer, chemical reactivity and kinetic/thermodynamic stability of a mol­ecule 1. The DFT study of the title compound revealed that the HOMO and LUMO are localized in the plane extending from the whole phenol ring to the pyridine ring and electron distribution of the HOMO-1, HOMO, LUMO and the LUMO+1 energy levels are shown in Fig. 4 ▸. Mol­ecular orbitals of HOMO contain both σ and π character, whereas HOMO-1 is dominated by π-orbital density. The LUMO is mainly composed of σ-density, while LUMO+1 is composed of both σ and π electron density. The HOMO–LUMO energy gap is very important for the chemical activity and explains the eventual charge transfer inter­action within the mol­ecule. The HOMO–LUMO gap was found to be 0.128907 a.u. and the frontier mol­ecular orbital energies, *E*_HOMO_ and *E*_LUMO_ were found to be as −0.19367 and −0.06476 a.u., respectively.

## Synthesis and crystallization   

The title compound was prepared from an equimolar mixture of 4-amino­phenyl­amino­methyl­phenol (0.50 g, 2.3 mmol) and pyridine-2-carbaldehyde (0.20 g, 2.30 mmol) in (50 ml) methanol. The yellow reaction mixture was stirred for 3 h at room temperature and solvent was evaporated to 5 ml. The resulting yellow solid was isolated by filtration, washed successively with a cold water and methanol mixture (10 ml) and hexane (20 ml). The compound was recrystallized from hot methanol, giving yellow plate-like crystals. Finally, the yellow solid was dried in a vacuum desiccator (yield 0.50 g, 70%; m.p. 446–448 K).

Spectroscopic data: UV–Vis (MeOH): λ_max_ nm (∊, *M* ^−1^ cm^−1^): 258 (13,000), 383 (16,000). IR (KBr, cm^−1^): ν(C=N) 1625, ν(N—H) 3265.

^1^H NMR (400 MHz, DMSO-*d*_6_): δ 8.6 (1H, *s*, CH=N), 7.4 (1H, *s*), 7.8 (1H, *t*, *J* = 8.4, 6.8 Hz), 8.0 (1H, *d*, *J* = 6.4 Hz), 8.5 (1H, *s*), 6.7 (2H, *d*, *J* = 6.0 Hz), 6.6 (2H, *d*, *J* = 6.4 Hz), 4.1 (2H, *s*), 7.1 (2H, *d*, *J* = 6.4 Hz), 7.2 (2H, *d*, *J* = 6.4 Hz), 9.3 (–OH), 6.5 (NH).

HRMS (ESI) *m*/*z* [*M* + H]^+^ calculated for C_19_H_17_N_3_O: 304.1444; found: 304.1455.

## Refinement   

Crystal data, data collection and structure refinement details are summarized in Table 3 ▸. The crystal diffracted very weakly beyond 20° in θ, and only *ca* 40% of the data can be considered to be observed; hence the large value for *R*_int_ of 0.122. The N—H and O—H H atoms were located in difference Fourier maps. The OH H atom was freely refined, while during refinement, the N- and C-bound H atoms were included in calculated positions and treated as riding, with N—H = 0.86 Å and C—H = 0.93 Å, and *U*_iso_(H) = 1.2*U*_eq_(C,N).

## Supplementary Material

Crystal structure: contains datablock(s) I, Global. DOI: 10.1107/S2056989018003043/su5421sup1.cif

Structure factors: contains datablock(s) I. DOI: 10.1107/S2056989018003043/su5421Isup2.hkl

CCDC reference: 1542988

Additional supporting information:  crystallographic information; 3D view; checkCIF report

## Figures and Tables

**Table 1:** Hydrogen-bond geometry (Å, °) *Cg* is the centroid of the pyridine ring, N3/C15-C19.

*D*—H⋯*A*	*D*—H	H⋯*A*	*D*⋯*A*	*D*—H⋯*A*
O1—H1⋯N3^i^	0.88 (2)	1.92 (2)	2.796 (3)	179 (3)
N1—H1*A*⋯O1^ii^	0.86	2.13	2.982 (3)	170
C7—H7*A*⋯*Cg* ^iii^	0.97	2.93	3.687 (3)	136

Symmetry codes: (i) 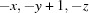; (ii) 
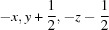
; (iii) 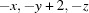.

**Table 2:** Comparison of selected geometric data for (I) (Å, °) from calculated (DFT) and X-ray data.

Bonds	X-ray	B3LYP/6–311G(d,p).
N1—C7	1.439 (3)	1.438
N1—C8	1.368 (3)	1.368
N2—C11	1.409 (3)	1.409
N2—C14	1.256 (3)	1.256
C1—O1	1.388 (3)	1.388
C4—C7	1.512 (3)	1.512
C14—C15	1.460 (3)	1.460
N1—C7—C4	112.3 (2)	112.28
C8—N1—C7	123.4 (2)	123.45
C11—N2—C14	121.5 (2)	121.54
N2—C14—C15	122.2 (3)	122.23
C4—C7—N1—C8	−166.3 (2)	−166.34
C15—C14—N2—C11	176.4 (2)	176.39

**Table 3:** Experimental details

Crystal data	
Chemical formula	C_19_H_17_N_3_O
*M*_r_	303.22
Crystal system, space group	Monoclinic, *P*2_1_/*c*
Temperature (K)	296
*a*, *b*, *c* (Å)	10.5652 (7), 7.9136 (6), 20.8153 (13)
β (°)	118.408 (4)
*V* (Å^3^)	1530.77 (19)
*Z*	4
Radiation type	Mo *K*α
μ (mm^−1^)	0.08
Crystal size (mm)	0.21 × 0.15 × 0.11

Data collection	
Diffractometer	Bruker SMART CCD area detector
Absorption correction	Multi-scan (*SADABS*; Bruker, 2012 ▸)
*T*_min_, *T*_max_	0.785, 0.856
No. of measured, independent and observed [*I* > 2σ(*I*)] reflections	17211, 2664, 1087
*R*_int_	0.122
(sin θ/λ)_max_ (Å^−1^)	0.595

Refinement	
*R*[*F* ^2^ > 2σ(*F* ^2^)], *wR*(*F* ^2^), *S*	0.040, 0.092, 0.73
No. of reflections	2664
No. of parameters	212
No. of restraints	7
H-atom treatment	H atoms treated by a mixture of independent and constrained refinement
Δρ_max_, Δρ_min_ (e Å^−3^)	0.14, −0.15

Computer programs: *SMART* and *SAINT* (Bruker, 2012 ▸), *SHELXT* (Sheldrick, 2015*a* ▸), *SHELXL2016* (Sheldrick, 2015*b* ▸), *ORTEP-3 for Windows* and *WinGX* (Farrugia, 2012 ▸), *Mercury* (Macrae *et al.*, 2008 ▸) and *PLATON* (Spek, 2009 ▸).

## supplementary crystallographic information

### Crystal data

**Table:**

C_19_H_17_N_3_O	*F*(000) = 640
*M**_r_* = 303.22	*D*_x_ = 1.316 Mg m^−^^3^
Monoclinic, *P*2_1_/*c*	Mo *K*α radiation, λ = 0.71073 Å
*a* = 10.5652 (7) Å	Cell parameters from 1114 reflections
*b* = 7.9136 (6) Å	θ = 2.8–18.2°
*c* = 20.8153 (13) Å	µ = 0.08 mm^−^^1^
β = 118.408 (4)°	*T* = 296 K
*V* = 1530.77 (19) Å^3^	Plate, yellow
*Z* = 4	0.21 × 0.15 × 0.11 mm

### Data collection

**Table:**

Bruker SMART CCD area detector diffractometer	2664 independent reflections
Radiation source: sealed tube	1087 reflections with *I* > 2σ(*I*)
Graphite monochromator	*R*_int_ = 0.122
phi and ω scans	θ_max_ = 25.0°, θ_min_ = 2.2°
Absorption correction: multi-scan (SADABS; Bruker, 2012)	*h* = −12→12
*T*_min_ = 0.785, *T*_max_ = 0.856	*k* = −9→9
17211 measured reflections	*l* = −24→23

### Refinement

**Table:**

Refinement on *F*^2^	Primary atom site location: structure-invariant direct methods
Least-squares matrix: full	Secondary atom site location: difference Fourier map
*R*[*F*^2^ > 2σ(*F*^2^)] = 0.040	Hydrogen site location: inferred from neighbouring sites
*wR*(*F*^2^) = 0.092	H atoms treated by a mixture of independent and constrained refinement
*S* = 0.73	*w* = 1/[σ^2^(*F*_o_^2^) + (0.035*P*)^2^] where *P* = (*F*_o_^2^ + 2*F*_c_^2^)/3
2664 reflections	(Δ/σ)_max_ < 0.001
212 parameters	Δρ_max_ = 0.14 e Å^−^^3^
7 restraints	Δρ_min_ = −0.15 e Å^−^^3^

### Special details

**Table:**

Geometry. All esds (except the esd in the dihedral angle between two l.s. planes) are estimated using the full covariance matrix. The cell esds are taken into account individually in the estimation of esds in distances, angles and torsion angles; correlations between esds in cell parameters are only used when they are defined by crystal symmetry. An approximate (isotropic) treatment of cell esds is used for estimating esds involving l.s. planes.

### Fractional atomic coordinates and isotropic or equivalent isotropic displacement parameters (Å^2^)

**Table:**

	*x*	*y*	*z*	*U*_iso_*/*U*_eq_	
O1	−0.2029 (2)	0.4843 (3)	−0.37245 (9)	0.0677 (6)	
N2	0.3840 (2)	0.9002 (3)	0.25128 (10)	0.0575 (6)	
N1	0.0654 (2)	0.8060 (2)	−0.05048 (10)	0.0584 (6)	
H1A	0.104122	0.846064	−0.075424	0.070*	
N3	0.4388 (2)	0.7338 (3)	0.42061 (11)	0.0551 (6)	
C6	−0.2709 (3)	0.6253 (3)	−0.29168 (13)	0.0618 (8)	
H6	−0.363931	0.638443	−0.329860	0.074*	
C1	−0.1675 (3)	0.5496 (3)	−0.30408 (13)	0.0536 (7)	
C2	−0.0284 (3)	0.5374 (3)	−0.24837 (13)	0.0569 (7)	
H2	0.042773	0.490422	−0.257010	0.068*	
C3	0.0050 (3)	0.5955 (3)	−0.17948 (12)	0.0560 (7)	
H3	0.099320	0.587513	−0.142046	0.067*	
C4	−0.0990 (3)	0.6653 (3)	−0.16507 (12)	0.0517 (7)	
C5	−0.2366 (3)	0.6822 (3)	−0.22213 (13)	0.0611 (8)	
H5	−0.307393	0.732180	−0.213979	0.073*	
C8	0.1329 (3)	0.8340 (3)	0.02322 (13)	0.0489 (7)	
C7	−0.0669 (3)	0.7130 (3)	−0.08846 (12)	0.0603 (8)	
H7A	−0.145423	0.781051	−0.090815	0.072*	
H7B	−0.060997	0.611176	−0.061241	0.072*	
C13	0.0849 (3)	0.7669 (3)	0.06958 (13)	0.0584 (7)	
H13	−0.001697	0.708081	0.050074	0.070*	
C12	0.1648 (3)	0.7869 (3)	0.14445 (13)	0.0579 (8)	
H12	0.131692	0.738651	0.174403	0.069*	
C11	0.2923 (3)	0.8763 (3)	0.17598 (13)	0.0508 (7)	
C10	0.3358 (3)	0.9513 (3)	0.12946 (13)	0.0567 (7)	
H10	0.419007	1.016616	0.149006	0.068*	
C9	0.2582 (3)	0.9308 (3)	0.05489 (13)	0.0554 (7)	
H9	0.290049	0.982488	0.025065	0.066*	
C14	0.3648 (3)	0.8223 (3)	0.29852 (13)	0.0590 (7)	
H14	0.285013	0.752151	0.283243	0.071*	
C15	0.4635 (3)	0.8382 (3)	0.37661 (13)	0.0515 (7)	
C16	0.5780 (3)	0.9501 (3)	0.40340 (13)	0.0598 (8)	
H16	0.592239	1.021323	0.371868	0.072*	
C17	0.6708 (3)	0.9544 (3)	0.47755 (14)	0.0647 (8)	
H17	0.748321	1.028674	0.496625	0.078*	
C18	0.6472 (3)	0.8476 (3)	0.52292 (14)	0.0616 (8)	
H18	0.708703	0.847287	0.573011	0.074*	
C19	0.5308 (3)	0.7418 (3)	0.49240 (14)	0.0594 (7)	
H19	0.514376	0.670992	0.523359	0.071*	
H1	−0.278 (3)	0.416 (4)	−0.3883 (16)	0.133 (15)*	

### Atomic displacement parameters (Å^2^)

**Table:**

	*U*^11^	*U*^22^	*U*^33^	*U*^12^	*U*^13^	*U*^23^
O1	0.0697 (15)	0.0891 (15)	0.0397 (11)	−0.0111 (13)	0.0222 (11)	−0.0157 (10)
N2	0.0604 (15)	0.0689 (15)	0.0356 (13)	−0.0049 (11)	0.0167 (12)	0.0004 (10)
N1	0.0669 (16)	0.0712 (16)	0.0359 (13)	−0.0119 (13)	0.0236 (12)	−0.0055 (11)
N3	0.0579 (15)	0.0626 (15)	0.0346 (13)	−0.0040 (12)	0.0137 (12)	−0.0020 (11)
C6	0.0483 (18)	0.083 (2)	0.0389 (17)	0.0012 (16)	0.0083 (15)	−0.0036 (14)
C1	0.063 (2)	0.0563 (19)	0.0415 (17)	−0.0068 (15)	0.0248 (16)	−0.0053 (13)
C2	0.0477 (19)	0.071 (2)	0.0448 (17)	0.0031 (15)	0.0160 (15)	−0.0046 (14)
C3	0.0480 (18)	0.0675 (19)	0.0385 (16)	0.0028 (15)	0.0091 (14)	−0.0027 (13)
C4	0.0574 (19)	0.0574 (18)	0.0360 (16)	0.0008 (14)	0.0187 (15)	−0.0017 (12)
C5	0.056 (2)	0.077 (2)	0.0458 (17)	0.0045 (15)	0.0208 (16)	−0.0058 (14)
C8	0.0572 (19)	0.0510 (18)	0.0332 (15)	0.0044 (14)	0.0172 (14)	0.0008 (12)
C7	0.0610 (19)	0.072 (2)	0.0436 (17)	−0.0006 (16)	0.0216 (15)	−0.0059 (14)
C13	0.0591 (18)	0.073 (2)	0.0391 (17)	−0.0132 (15)	0.0198 (15)	−0.0047 (13)
C12	0.0629 (19)	0.068 (2)	0.0411 (17)	−0.0106 (16)	0.0237 (15)	−0.0001 (13)
C11	0.0574 (19)	0.0553 (18)	0.0352 (16)	−0.0046 (14)	0.0182 (15)	0.0004 (13)
C10	0.0584 (19)	0.0597 (19)	0.0475 (17)	−0.0085 (14)	0.0216 (16)	−0.0041 (13)
C9	0.063 (2)	0.062 (2)	0.0435 (17)	−0.0077 (15)	0.0272 (16)	−0.0019 (13)
C14	0.0554 (17)	0.0665 (19)	0.0426 (17)	−0.0065 (14)	0.0130 (14)	−0.0040 (13)
C15	0.0539 (18)	0.0554 (19)	0.0398 (16)	0.0045 (15)	0.0179 (15)	−0.0044 (13)
C16	0.066 (2)	0.065 (2)	0.0465 (18)	−0.0075 (16)	0.0251 (16)	−0.0048 (14)
C17	0.060 (2)	0.068 (2)	0.0556 (19)	−0.0099 (15)	0.0192 (17)	−0.0072 (15)
C18	0.061 (2)	0.067 (2)	0.0398 (16)	0.0021 (16)	0.0099 (15)	−0.0047 (15)
C19	0.069 (2)	0.0624 (19)	0.0396 (18)	−0.0046 (17)	0.0198 (16)	−0.0007 (13)

### Geometric parameters (Å, º)

**Table:**

O1—C1	1.388 (3)	C8—C9	1.394 (3)
O1—H1	0.879 (17)	C7—H7A	0.9700
N2—C14	1.256 (3)	C7—H7B	0.9700
N2—C11	1.409 (3)	C13—C12	1.384 (3)
N1—C8	1.368 (3)	C13—H13	0.9300
N1—C7	1.439 (3)	C12—C11	1.380 (3)
N1—H1A	0.8600	C12—H12	0.9300
N3—C19	1.341 (3)	C11—C10	1.387 (3)
N3—C15	1.347 (3)	C10—C9	1.377 (3)
C6—C1	1.374 (3)	C10—H10	0.9300
C6—C5	1.390 (3)	C9—H9	0.9300
C6—H6	0.9300	C14—C15	1.460 (3)
C1—C2	1.376 (3)	C14—H14	0.9300
C2—C3	1.382 (3)	C15—C16	1.385 (3)
C2—H2	0.9300	C16—C17	1.380 (3)
C3—C4	1.384 (3)	C16—H16	0.9300
C3—H3	0.9300	C17—C18	1.376 (3)
C4—C5	1.378 (3)	C17—H17	0.9300
C4—C7	1.512 (3)	C18—C19	1.369 (3)
C5—H5	0.9300	C18—H18	0.9300
C8—C13	1.391 (3)	C19—H19	0.9300
			
C1—O1—H1	112 (2)	C12—C13—C8	120.6 (2)
C14—N2—C11	121.5 (2)	C12—C13—H13	119.7
C8—N1—C7	123.4 (2)	C8—C13—H13	119.7
C8—N1—H1A	118.3	C11—C12—C13	121.9 (2)
C7—N1—H1A	118.3	C11—C12—H12	119.0
C19—N3—C15	117.2 (2)	C13—C12—H12	119.0
C1—C6—C5	120.0 (2)	C12—C11—C10	117.4 (2)
C1—C6—H6	120.0	C12—C11—N2	126.7 (2)
C5—C6—H6	120.0	C10—C11—N2	116.0 (2)
C6—C1—C2	119.9 (2)	C9—C10—C11	121.2 (2)
C6—C1—O1	120.3 (2)	C9—C10—H10	119.4
C2—C1—O1	119.8 (3)	C11—C10—H10	119.4
C1—C2—C3	119.6 (2)	C10—C9—C8	121.3 (2)
C1—C2—H2	120.2	C10—C9—H9	119.3
C3—C2—H2	120.2	C8—C9—H9	119.3
C2—C3—C4	121.3 (2)	N2—C14—C15	122.2 (3)
C2—C3—H3	119.3	N2—C14—H14	118.9
C4—C3—H3	119.3	C15—C14—H14	118.9
C5—C4—C3	118.2 (2)	N3—C15—C16	122.2 (2)
C5—C4—C7	120.0 (2)	N3—C15—C14	115.9 (2)
C3—C4—C7	121.7 (2)	C16—C15—C14	121.9 (2)
C4—C5—C6	120.8 (2)	C17—C16—C15	119.1 (2)
C4—C5—H5	119.6	C17—C16—H16	120.5
C6—C5—H5	119.6	C15—C16—H16	120.5
N1—C8—C13	123.3 (2)	C18—C17—C16	119.2 (3)
N1—C8—C9	119.4 (2)	C18—C17—H17	120.4
C13—C8—C9	117.3 (2)	C16—C17—H17	120.4
N1—C7—C4	112.3 (2)	C19—C18—C17	118.3 (2)
N1—C7—H7A	109.2	C19—C18—H18	120.9
C4—C7—H7A	109.2	C17—C18—H18	120.9
N1—C7—H7B	109.2	N3—C19—C18	124.0 (2)
C4—C7—H7B	109.2	N3—C19—H19	118.0
H7A—C7—H7B	107.9	C18—C19—H19	118.0
			
C5—C6—C1—C2	−3.1 (4)	C13—C12—C11—N2	−178.0 (2)
C5—C6—C1—O1	176.5 (2)	C14—N2—C11—C12	8.6 (4)
C6—C1—C2—C3	2.6 (4)	C14—N2—C11—C10	−171.5 (2)
O1—C1—C2—C3	−177.0 (2)	C12—C11—C10—C9	−2.9 (4)
C1—C2—C3—C4	0.3 (4)	N2—C11—C10—C9	177.2 (2)
C2—C3—C4—C5	−2.6 (4)	C11—C10—C9—C8	0.0 (4)
C2—C3—C4—C7	173.8 (2)	N1—C8—C9—C10	−174.9 (2)
C3—C4—C5—C6	2.1 (4)	C13—C8—C9—C10	3.5 (4)
C7—C4—C5—C6	−174.3 (2)	C11—N2—C14—C15	176.4 (2)
C1—C6—C5—C4	0.7 (4)	C19—N3—C15—C16	−0.2 (3)
C7—N1—C8—C13	3.9 (4)	C19—N3—C15—C14	177.8 (2)
C7—N1—C8—C9	−177.7 (2)	N2—C14—C15—N3	−173.0 (2)
C8—N1—C7—C4	−166.3 (2)	N2—C14—C15—C16	5.0 (4)
C5—C4—C7—N1	−137.0 (2)	N3—C15—C16—C17	0.4 (4)
C3—C4—C7—N1	46.7 (3)	C14—C15—C16—C17	−177.5 (2)
N1—C8—C13—C12	174.1 (2)	C15—C16—C17—C18	0.0 (4)
C9—C8—C13—C12	−4.3 (4)	C16—C17—C18—C19	−0.7 (4)
C8—C13—C12—C11	1.5 (4)	C15—N3—C19—C18	−0.5 (4)
C13—C12—C11—C10	2.1 (4)	C17—C18—C19—N3	1.0 (4)

### Hydrogen-bond geometry (Å, º)

Cg is the centroid of the pyridine ring, N3/C15-C19.

**Table:**

*D*—H···*A*	*D*—H	H···*A*	*D*···*A*	*D*—H···*A*
O1—H1···N3^i^	0.88 (2)	1.92 (2)	2.796 (3)	179 (3)
N1—H1*A*···O1^ii^	0.86	2.13	2.982 (3)	170
C7—H7*A*···*Cg*^iii^	0.97	2.93	3.687 (3)	136

Symmetry codes: (i) −*x*, −*y*+1, −*z*; (ii) −*x*, *y*+1/2, −*z*−1/2; (iii) −*x*, −*y*+2, −*z*.
